# Impact of Different Volumes of Pericapsular Nerve Group Block on Pain During Spinal Anesthesia Positioning and Postoperative Opioid Requirements in Femoral Fracture Surgeries; Randomized Prospective Study

**DOI:** 10.2147/JPR.S468863

**Published:** 2024-09-18

**Authors:** Gamze Ertaş, Hamiyet Şenol Çakmak, Sevda Akdeniz, Alparslan Yurtbay, Ebru Polat, Yavuz Yigit, Nezih Sertöz, Serkan Tulgar

**Affiliations:** 1 Department of Anesthesiology, Samsun University Faculty of Medicine, Samsun Training and Research Hospital, Samsun, Turkiye; 2 Department of Orthopedic Surgery, Samsun University Faculty of Medicine, Samsun Training and Research Hospital, Samsun, Turkiye; 3 Department of Emergency Medicine, Hamad General Hospital, Hamad Medical Corporation, Doha, Qatar; 4 Blizard Institute, Queen Mary University, London, UK; 5 Department of Anesthesiology and Reanimation, Ege University, Faculty of Medicine, İzmir, Turkiye

**Keywords:** hip fracture surgery, pericapsular nerve group block, PENG, spinal anesthesia positioning, postoperative analgesia, local anesthetic volume

## Abstract

**Introduction:**

Hip fracture surgeries in patients present significant challenges, particularly in managing pain during spinal anesthesia positioning. The Pericapsular Nerve Group Block (PENG) has shown promise in addressing this issue, but the ideal volume of local anesthetic for PENG is still uncertain. In our study, we aimed to analyze the effects of administering PENG block with two different volumes on analgesic quality for patients undergoing hip fracture surgery.

**Methods:**

In this prospective, randomized controlled trial, the effects of administering a PENG block with 20 mL versus 30 mL of local anesthetic in patients undergoing hip fracture surgery under spinal anesthesia were compared. The primary outcome was pain during spinal anesthesia positioning, and secondary outcomes included postoperative pain scores and opioid consumption.

**Results:**

A total of 60 patients were analyzed, with 30 in each group. Critical parameters such as the time of spinal anesthesia administration and the satisfaction of the anesthesiologist showed no significant differences (p=0.918; p=0.741, respectively). NRS scores recorded before, during, and after the positioning for spinal anesthesia exhibited similar patterns (p=0.290; p=0.247; p=0.288, respectively). The cumulative opioid requirements did not exhibit a statistically significant difference at 24 hours (p = 0.098). Quadriceps weakness was significantly more in the PENG-30 group 6 hours after surgery but had recovered by the 9th hour (p= 0.004).

**Conclusion:**

In patients undergoing hip fracture surgery, the effects of applying the PENG block with 20 mL or 30 mL of local anesthetic are comparable in terms of positioning for spinal anesthesia and postoperative analgesic requirements.

## Introduction

Hip fracture surgeries in elderly patients are commonly performed orthopedic procedures and are associated with high mortality and morbidity rates.^1^,^2^ Clinicians consider the patient’s comorbidities when determining anesthesia management, choosing between general or neuraxial anesthesia techniques.^3^ In certain cases, plexus blocks or alternative regional anesthesia techniques may be considered based on factors such as the type of fracture, surgical incision site, and the intended sensory block area.^4^ Regardless of the chosen anesthesia management, patients are expected to endure considerable pain during activities like transfer and spinal positioning for spinal anesthesia. Regional anesthesia techniques can be utilized as a proactive strategy to address this pain.

Fascia iliaca compartment blocks, performed with both suprainguinal and infrainguinal approaches, are commonly used in these patients to prevent positioning pain during spinal anesthesia.^5^,^6^ The Pericapsular Nerve Group Block (PENG) is a relatively newer and increasingly popular technique that specifically targets the anterior surface of the hip capsule.^7^

The PENG block, performed under ultrasound (US) guidance, was introduced by Giron-Arango et al in 2018 with the aim of blocking the articular branch of the femoral nerve, the articular branch of the obturator nerve, and the accessory obturator nerve. PENG block has been used for managing acute pain in hip fractures^8^ and has later been employed for addressing both positional and postoperative pain during subsequent periods.^9^,^10^ There have been anecdotal reports and hypotheses which suggest that depending on different volumes administered into PENG block may present varying effects. These accounts indicate that higher volumes could potentially result in more extensive spread at times resembling effects similar to lumbar plexus block as demonstrated by anatomical studies and anecdotal case reports.^11–14^ PENG block, typically administered with 15–20 mL, aims to block the pericapsular nerve group and relieve hip joint pain. Higher volumes, such as 30 mL, may extend to the lumbar plexus, providing broader analgesia.^12^,^13^ This study investigates the clinical differences in hip fracture patients when using 20 mL versus 30 mL for the PENG block.

In this study, our main objective was to examine the impact of administering PENG block with two different volumes on the analgesic quality for patients undergoing hip fracture surgery. The primary outcome aimed to assess how PENG block, given at varying volumes, affected pain experienced during spinal anesthesia positioning. Secondary outcomes included the postoperative pain scores (measured using the numerical rating scale; NRS) and opioid requirements within the initial 24 hours across different groups.

## Materials and Methods

### Study Design

**Figure 1:**
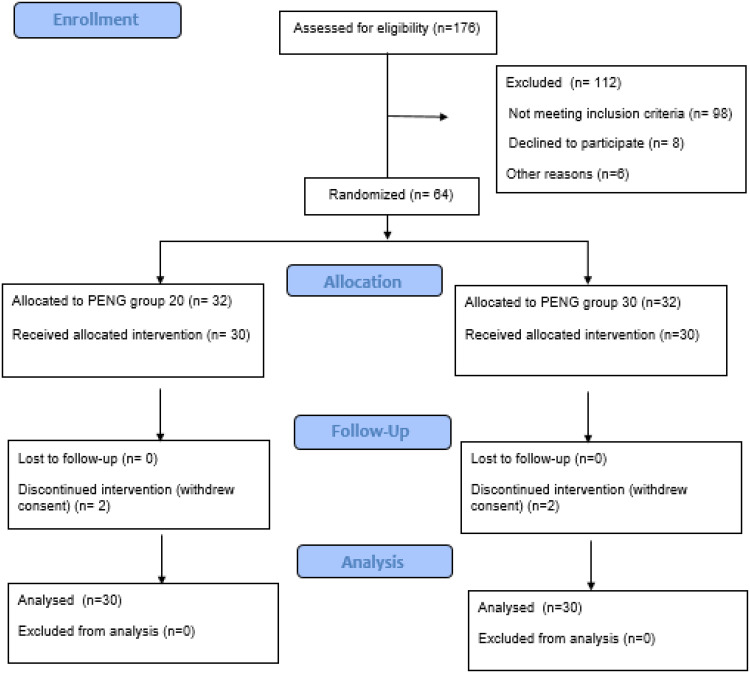
Flow chart of study.

**Abbreviation**: PENG, Pericapsular Nerve Group Block.

This prospective, randomized, controlled study received approval from the Local Ethics Committee (Samsun Ondokuz Mayıs University - Clinical Research Ethics Committee: 2022/62), Ministry of Health (Approval code: 22/AKD:63), and was registered on clinicaltrials.org (NCT05358587, Registration date: 20/04/2022). The research, conducted in the Department of Anesthesiology at Samsun University Training and Research Hospital, spanned from May 2022 to January 2024. The study’s adherence to the principles of the Declaration of Helsinki was ensured, and written informed consent was obtained from all participants. The CONSORT checklist for the study is available in Figure 1.

The study included patients aged between 35 and 90 years, scheduled for hip fracture surgery under spinal anesthesia, with ASA (American Society of Anesthesiologists) classifications I–III. Patients with contraindications to central nerve blocks, dementia, psychiatric and neurological disorders leading to impaired consciousness, multiple fractures, or falling in the ASA IV–V categories were excluded. Moreover, participants who declined to provide voluntary informed consent or indicated disinterest in taking part were not included in the study.

### Grouping and Randomisation

The patients were randomized into two groups, labelled as Group I and Group II, one hour before the surgical procedure using a sealed envelope method. Group I received 20 mL of local anesthetic, while Group II received 30 mL of local anesthetic. The general recommendation for PENG application is a volume range of 15–20 mL. Therefore, we selected 20 mL as the standard volume for our study’s normal volume group. Previous literature^12^ defined high volume as 30 mL, which guided our choice. The randomization and block procedures were carried out by the same anesthesiologist (ST). The administering anesthetist had no involvement in data collection or analysis.

All patients were admitted to the block room prior the surgical procedure and then transferred to the operating room. An uninformed assessor, unaware of the interventions or groups, was brought to the room just before the “placement of spinal anesthesia.” This uninformed assessor collected and evaluated all data, including spinal anesthesia placement pain, postoperative pain, and opioid requirements.

### Interventions

Patients were taken to the block room and procedures were performed under basic monitoring. After securing vascular access, routine anesthesia monitoring was conducted, and oxygen was administered via a nasal cannula. All block procedures were performed by a single anesthesiologist (ST) in the block room. Following proper skin antisepsis, the block was carried out using a convex ultrasound (USG) transducer (3–5 MHz, Esaote MyLab 30 gold, France) and a 21 G 10 cm block needle (Pajunk, sonoplex, B. Braun, Bethlehem, PA) with an in-plane technique. The USG transducer was positioned over the anterior superior iliac spine in the transverse plane. It was then shifted caudally to identify the spina iliaca anterior inferior, being slightly oblique in the superolateral and inferomedial directions. The transducer provided a comprehensive view of structures such as the femoral artery, femoral nerve, iliacus muscle, psoas tendon, iliopubic eminence, and spina iliaca anterior inferior. Particular attention was paid to exclude the hip joint and femoral head from the visual field. The needle was advanced in-plane from lateral to medial, directing the needle tip between the iliopsoas tendon (IPT) and iliopubic eminence (IPE). Subsequently, 20 mL of 0.25% bupivacaine was administered to patients in Group PENG-20, and 30 mL of 0.25% bupivacaine was administered to patients in Group PENG-30.

### Spinal Anesthesia Management

Spinal anesthesia was applied 30 minutes after the end of the PENG block performance. All patients received spinal anesthesia in a sitting position under sterile conditions, using a 25 G spinal needle, for the administration of 10–12.5 mg of hyperbaric bupivacaine at the L4-L5 interspinous levels. We chose the sitting position for our study on neuraxial anesthesia positioning pain in hip fracture patients, aligning with most literature that investigates factors affecting spinal anesthesia positioning pain. While lateral position and spinal hemiblock were alternatives, we opted for sitting to ensure study homogenization. No additives, including intrathecal morphine, were used. Following the intrathecal injection, patients were immediately placed in the supine position, and the side to be operated on was maintained at a 30-degree angle downward for 5 minutes. After confirming the success of spinal anesthesia through the pinprick test, the surgical procedure was initiated.

### Pain Evaluation, Postoperative Analgesia and Quality Evaluations

The Numeric Rating Scale (NRS) was utilized for pain intensity assessment. NRS is a segmented numerical version of the visual analog scale (VAS), representing a one-dimensional measurement of adult pain intensity. Participants select a whole number that best represents their level of pain, ranging from 0 to 10 on an 11-point numerical scale. This ranges from “0” for no pain at one end (eg, “no pain at all”) to ‘10’ for the worst imaginable or “as bad as it can be” at the other end.

The follow-up time points were as follows:

*Preoperative NRS*: NRS at rest before the intervention.

*Pre-Positioning NRS*: NRS at rest immediately before positioning.

*Positioning NRS*: NRS at the moment of positioning within the first 30 minutes after the PENG block.

Post-Positioning NRS: NRS at rest in the supine position after spinal anesthesia.

Additionally, NRS was measured at rest postoperatively at the 3rd, 6th, 12th, 18th, and 24th hours.

All patients were given a 1 g intravenous dose of paracetamol in the recovery room immediately after surgery, followed by subsequent doses every 6 hours for the first 24-hour period. Intravenous patient-controlled analgesia was initiated for all patients in the recovery room with specific instructions to use the PCA device intravenously when the Numeric Rating Scale for pain intensity reached ≥ 4/10. Patient follow-up extended for 24 hours, and the PCA device was discontinued after this period.

### Outcomes Measurements

The primary outcome measure was the Numeric Rating Scale (NRS) scores during positioning for spinal anesthesia (*Positioning NRS*).

Secondary outcomes included:

*Spinal Anesthesia Application Time*: Measured from the initiation of positioning maneuvers until the removal of the spinal needle.

*Quality of Patient Positioning*: Evaluated by the anesthesiologist administering the spinal anesthesia. Patient positioning was rated as “unsatisfactory”, “satisfactory”, “good”, or “excellent”, assigning 0-1-2-3 points, respectively.

*Analgesic Consumption and the first opioid requirement time*: Cumulative morphine consumption measured via Patient Controlled Analgesia (PCA) throughout the day was noted, and time to first opioid request was also recorded.

*Quality of Recovery 15 (QoR 15) Score*: A score including 15 questions assessing the patient’s recovery quality.

*Quadriceps Weakness*: Graded on a 3-point scale: normal strength = 0 points (extension against resistance); paresis = 1 point (extension against gravity but not against resistance); and paralysis = 2 points (no extension).

### Sample Size and Statistical Analysis

To determine the sample size, a preliminary study was conducted involving five participants in each group. In this initial investigation, the observed maximum NRS score during positioning for spinal anesthesia was 3.4 ± 1.14 for Group PENG-20 and 2.4 ± 0.89 for Group PENG-30. Based on these findings, along with an alpha of 5%, beta of 10%, and a power of 95%, a minimum sample size of 28 patients per group was calculated. To accommodate potential dropouts, the decision was made to include 30 patients in each group.

Data were statistically analyzed using SPSS for Windows, Version 16.0 (SPSS Inc, Chicago, USA). The normal distribution was assessed via the Kolmogorov–Smirnov test. Continuous variables were presented as mean ± standard deviation or median (25th–75th percentiles). The *t*-test was employed for continuous variables with equal variance, while the Mann Whitney *U*-test was used for non-normally distributed data. Chi-square and Fisher’s exact tests compared ratios and categorical data. Kaplan-Meier analysis and the Wilcoxon test assessed time to first analgesia requirement. Statistical significance was set at p < 0.05, except for postoperative NRS scores, where significance after Bonferroni correction was considered at p < 0.008 to avoid potential issues with multiple comparisons.

## Results

Eligibility assessments were conducted on a cohort of 176 patients, leading to the exclusion of 112 individuals and subsequent randomization of 64 patients into two distinct groups, as visually represented in the CONSORT Flow diagram (Figure 1). The final analysis focused on 30 patients from each group, ensuring a comprehensive evaluation.

**Figure 2:**
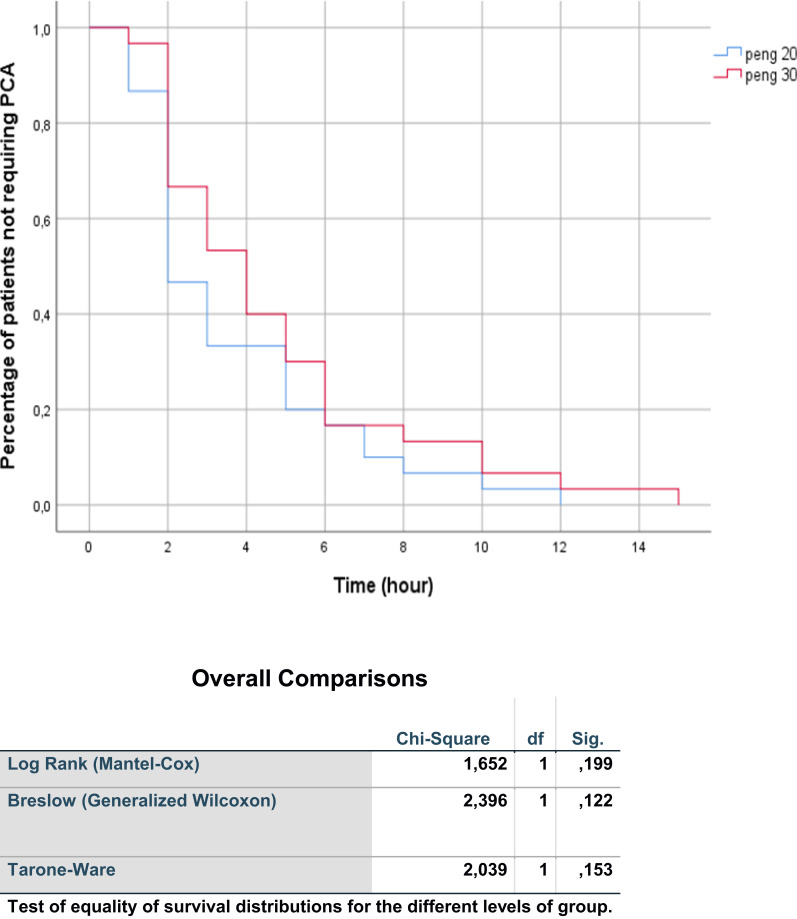
Kaplan-Meier graph of the time to first analgesic requirement according to group.

**Abbreviation**: PCA, Patient Controlled Analgesia.

The demographic characteristics of the participants are comprehensively outlined in Table 1, demonstrating a notable similarity between the two groups. Critical parameters such as the time of spinal anesthesia administration and the satisfaction of the anesthesiologist showed no significant differences (p=0.918; p=0.741, respectively). Baseline Numeric Rating Scale (NRS) scores were consistently comparable between the groups (p=0.769). Additionally, NRS scores recorded before, during, and after the positioning for spinal anesthesia exhibited similar patterns (p=0.290; p=0.247; p=0.288, respectively). The first analgesic requirement times were 3.66±2.79 hours in the PENG-20 group and 4.7±3.35 hours in the PENG-30 group, and this difference was not statistically significant (p=0.196). Figure 2 shows a Kaplan-Meier graph of the time to first analgesic requirement according to group (Log rank: 1.652, p:0.199).

**Table 1:** Descriptive Characteristics and Analgesia Requirements of Patients

	PENG-20 (n:30)	PENG-30 (n:30)	p
Age (years)	74.53±9.70	71.53±8.35	0.204
Gender F/M (n)	23/7	19/11	0.259
Height (cm)	157.83±16.15	162.5±9.38	0.176
Weight (kg)	75.06±17.84	76.96±16.44	0.669
Body Mass Index (kg/m^2)^	28.89±4.88	28.04±4.08	0.467
Spinal Anesthesia Performance Time (min)	4.36±1.09	4.33±1.18	0.918
Surgical time(min)	50.66±12.84	51.33±12.65	0.839
Anesthesiologist satisfaction	3.4±0.67	3.46±0.73	0.741
Time of the First Opioid Requirement (hours)	3.66±2.79	4.7±3.35	0.196

**Abbreviation**: PENG, Pericapsular Nerve Group Block.

Table 2 presents scores measured at specific time frames and cumulative opioid requirements. When comparing postoperative NRS scores between groups at the 3rd, 6th, 9th, 12th, 18th, and 24th hours, both groups were similar to each other (p<0.008). Cumulative opioid consumption was only lower in the PENG-30 group than the PENG-20 group at the 3rd hour (1.73±1.85 mg vs 0.63±1.18 mg, p=0.008), and in subsequent measurements, there were no significant differences between the groups (p>0.05). At the 24th hour, morphine consumption in the PENG-20 group was 12.66±6.84 mg, while in the PENG-30 group, it was 9.96±5.70 mg, and this difference was statistically insignificant (p=0.098).

**Table 2:** Cumulative Morphine Consumption (Mg)

	PENG 20	PENG 30	p
3rd hour	1 (0–3)	0 (0–1)	0.008
6th hour	3 (2–5)	2 (1–3)	0.082
9th hour	6 (3–7.75)	4 (3–6)	0.116
12th hour	7.5 (5–10)	5.5 (3.25–8.5)	0.080
18th hour	10.5 (5–13.75)	7 (5–11)	0.085
24th hour	12 (6.75–15.75)	7 (5–11)	0.102

**Abbreviation**: PENG, Pericapsular Nerve Group Block.

NRS scores for positioning and postoperative periods did not show any significant difference (Tables 3 and 4).

**Table 3:** Postoperative NRS Scores

	PENG 20	PENG 30	P
3rd hour	2.5 (0.25–4)	0.5 (0–2)	0.024
6th hour	3 (2–4)	3 (2–4)	0.871
9th hour	3.5 (2.25–4)	3 (2–4)	0.477
12th hour	2.5 (2–4)	3 (2–3.75)	0.783
18th hour	3 (1.25–4)	3 (2–3.75)	0.856
24th hour	2.5 (2–3.75)	3 (2–4)	0.744

**Abbreviation**: NRS, Numeric Rating Scale.

**Table 4:** NRS Scores During Position

	PENG 20	PENG 30	P
Preop NRS	5 (4–8)	7 (4–8)	0.769
Prepositioning NRS	2 (1–3.75)	3 (2–4)	0.290
Positioning NRS	3 (2–4.75)	4 (2.25–5)	0.247
Post positioning NRS	2 (0–2)	2 (0–3)	0.288

**Abbreviation**: Numeric Rating Scale.

There was a significant increase in quadriceps weakness among those who received 30 mL of local anesthetic with PENG at the 6th hour after surgery, but it resolved by the 9th hour (p=0.004) (Table 5).

**Table 5:** QoR-15 Scores and Quadriceps Weakness

	PENG 20	PENG 30	P
QoR-15 Scores	107.03±11.68	112.06±11.49	0.098
6th hour: Quadriceps weakness 0/(1/2)	21/(9/0)	10/(15/5)	0.004
9th hour Quadriceps weakness 0/(1/2)	30/(0/0)	27/(3/0)	

**Abbreviation**: QoR, Quality of Recovery.

## Discussion

In our study, we found that the preoperative application of PENG block with 20 mL and 30 mL local anesthetic in patients undergoing hip hemiarthroplasty had similar effects on spinal anesthesia positioning pain. Additionally, both volumes demonstrated comparable effects on postoperative analgesia scores and opioid requirements. However, there was a noticeable increase in quadriceps weakness among those who received 30 mL of local anesthetic with PENG at the 6th hour after surgery.

With the effective integration of ultrasound technology into clinical practice, regional anesthesia techniques have evolved, shifting towards interfascial plane or field blocks in addition to selective nerve or plexus blocks.^15^ Interfascial plane blocks and field blocks such as PENG have become commonly used in hip surgeries due to their potential benefits.

In hip fracture surgeries, neuraxial methods are commonly preferred for anesthesia. Nonetheless, managing positioning pain during the procedure can be highly distressing for the patient and presents a challenge for the anesthetist to address. Several controlled studies have investigated the use of the PENG block, initially designed to manage hip fracture pain, as a strategy to reduce or prevent positioning pain associated with spinal anesthesia in patients undergoing hip fracture surgery. In most of these studies, 15–20 mL of local anesthetic has been used.^9^,^16^,^17^ Furthermore, comparisons between the analgesic effects of this technique on positioning pain during spinal anesthesia for hip surgery and the suprainguinal fascia iliaca block have indicated that the PENG block is superior.^5^ However, although differences in the spread of the PENG block with two different volumes have been demonstrated cadaverically,^11^ as of our knowledge, there has not been a comparative study reporting its effects on positioning pain, postoperative pain scores, and analgesic consumption, and our study has investigated this aspect.

In some anecdotal writings, it has been reported that the PENG block, when applied with 30 mL or more of local anesthetic, may exert an effect similar to lumbar plexus block, and in some selected cases, it may even be used as an anesthetic method.^12^,^13^,^18^

However, as in other types of interfascial plane blocks and field blocks, in the PENG block as well, various factors beyond the volume effect on local anesthetic spread can play a role, such as the continuity of the fascial plane, the presence or absence of bone and tissue integrity, the patient’s age, muscle tone, injection pressure, anatomical variations, and more.^19^

Anatomically, the anterior surface of the hip capsule is innervated by the pericapsular nerve group, which can be blocked using the PENG block.^11^

On the other hand, the back of the hip joint receives nerves mainly from the sacral plexus, including branches like superior gluteal nerve, inferior gluteal nerve, sciatic nerve and its articular branches, as well as nerve to quadratus femoris muscle and cutaneous nerve of thigh.^20^ While blocking these nerves and branches may enhance the quality of analgesia, our study did not focus on this area, and it remains an anatomical region that needs consideration as a future insight.

In our study, we found that increasing the volume did not lead to additional benefits in terms of the primary outcome, which was spinal anesthesia positioning pain. Additionally, NRS scores were similar across all time frames. The only exception was at the 3rd hour when opioid consumption was noticeably higher in the PENG-20 group, and this difference proved to be statistically significant. However, we do not consider this clinically significant, as it may be directly related to the timing of the initial analgesic requirement. While no statistical significance was observed regarding other parameters, it’s worth mentioning that the time to first analgesia seemed marginally shorter in the PENG-20 group. This observation possibly contributed to the disparity in opioid consumption between groups. Opioid consumption remained similar in subsequent time frames. Previous studies reported a relatively longer time to first analgesia,^9^ this discrepancy could arise from methodological differences or diverse surgical procedures. In our study, we documented the first postoperative analgesia time from the conclusion of surgery. When considering parameters such as the interval from the end of the PENG block to spinal anesthesia, spinal anesthesia onset time, surgical duration, and others, it becomes evident that the duration of PENG block’s effect is relatively prolonged. However, given that the PENG block has minimal or no impact on cutaneous pain, the data regarding the first analgesia time obtained in our study can be deemed reasonable.

High-volume PENG application is often avoided due to the risk of prolonged motor weakness. In our study, we observed a higher incidence of quadriceps weakness in the PENG-30 group at 6 hours postoperatively. However, by the 9th hour, nearly all patients in both groups had regained full motor strength. For patients deemed suitable for early mobilization within the initial six hours, opting for a low-volume PENG application appears to be a more judicious choice. The potential mechanisms contributing to this motor weakness have been explored in prior hypothetical discussions, with the prevailing hypothesis involving the infiltration of local anesthetic to the femoral nerve through the iliacus muscle. Our findings align with this hypothesis, prompting us to exercise caution in ardently endorsing the high-volume PENG approach, which, in our investigation, did not yield discernible additional analgesic benefits.

Our study has some limitations. Firstly, we could have objectively measured motor strength using a dynamometer; however, due to the likelihood of patients with hip fractures avoiding movement because of pain, obtaining baseline values would have been challenging, leading to statistical difficulties in analysis. Therefore, comparing different volumes for motor weakness with voluntary movements would be beneficial. Another limitation is the absence of a control group. We designed and conducted our methodology as a comparative study, avoiding the application of a technique previously shown to reduce positioning pain in hip fracture patients, considering it as subjecting patients to pain, and thus refrained from implementing it. Furthermore, while we do not categorize it as a limitation, an essential consideration is that the dosage of local anesthetic administered in two different volumes will vary. This variation may lead to differences in the systemic absorption of bupivacaine and plasma levels between the groups. We were unable to devise a study design that completely eliminates the direct systemic effects of local anesthetics; one potential approach could involve diluting the same dose into two different volumes. However, such a modification might influence the rate of absorption through fascial and muscular tissues. Moreover, as with any fascial plane block, there exists a potential for myotoxicity with PENG. Given the varying distribution areas due to the different volumes used, the nature and extent of myotoxicity may differ accordingly. While we could have assessed this indirectly through markers like serum creatine phosphokinase levels, we acknowledge that not doing so represents another limitation of our study.

## Conclusion

Our study revealed that in patients undergoing hip fracture surgery under spinal anesthesia, both 20 mL and 30 mL local anesthetic applications of PENG block demonstrated similar efficacy in managing spinal anesthesia positioning pain and exhibited comparable analgesic effects in the postoperative period. We found that applying PENG with a higher volume did not provide additional benefits, leading us to advise against increasing the volume. However, applying PENG with 30 mL is associated with increased quadriceps weakness at the 6th postoperative hour compared to 20 mL, potentially complicating early mobilization.

## Data Availability

The datasets used and/or analysed during the current study available from the corresponding author on reasonable request.
